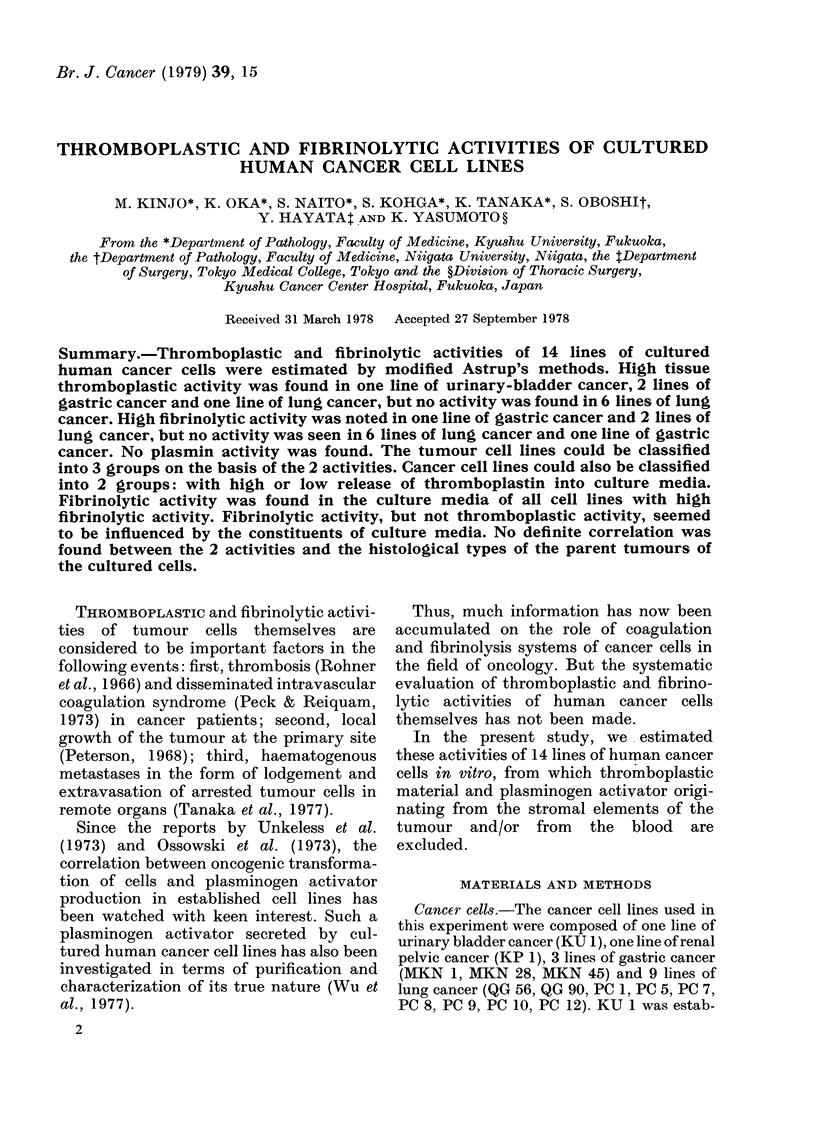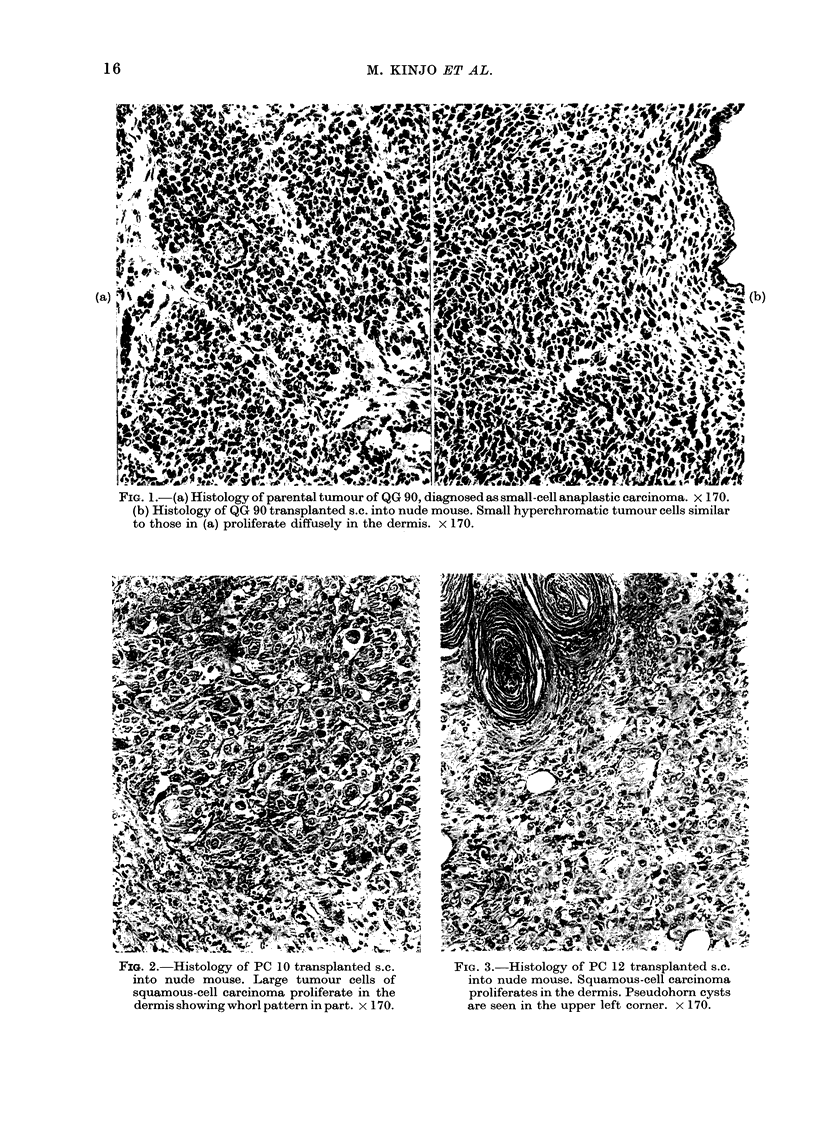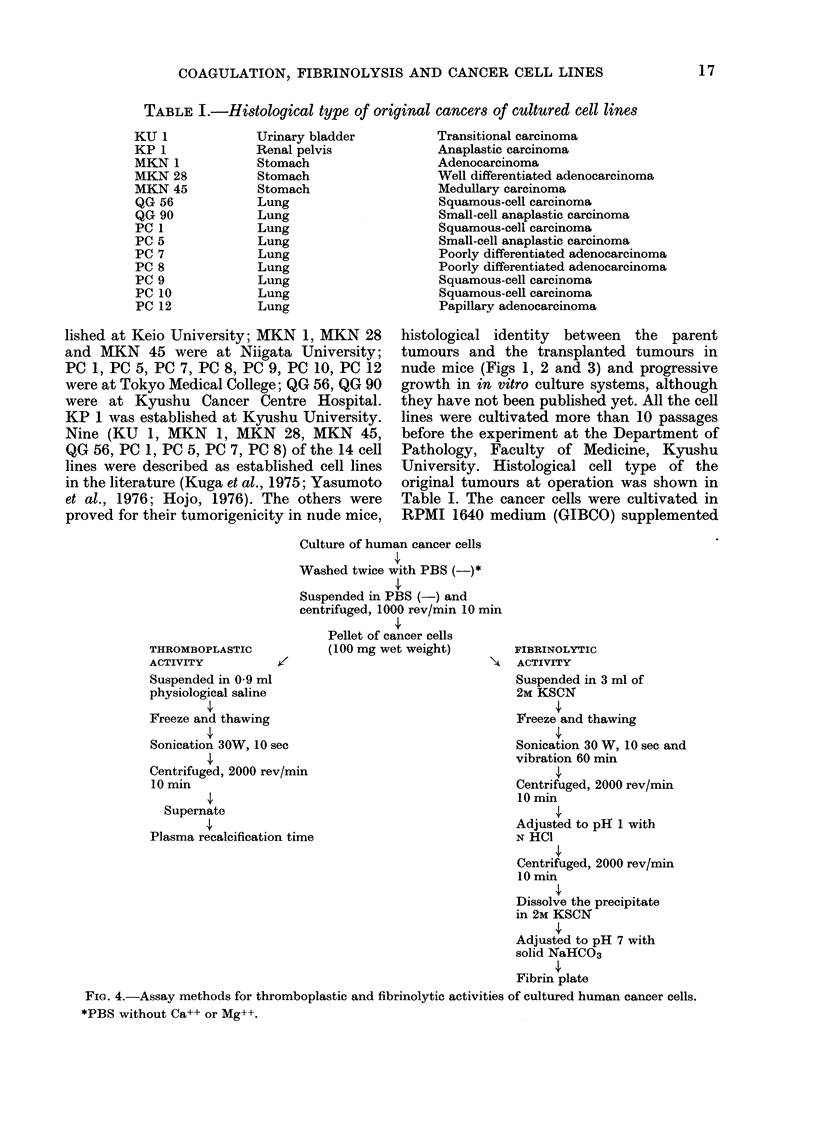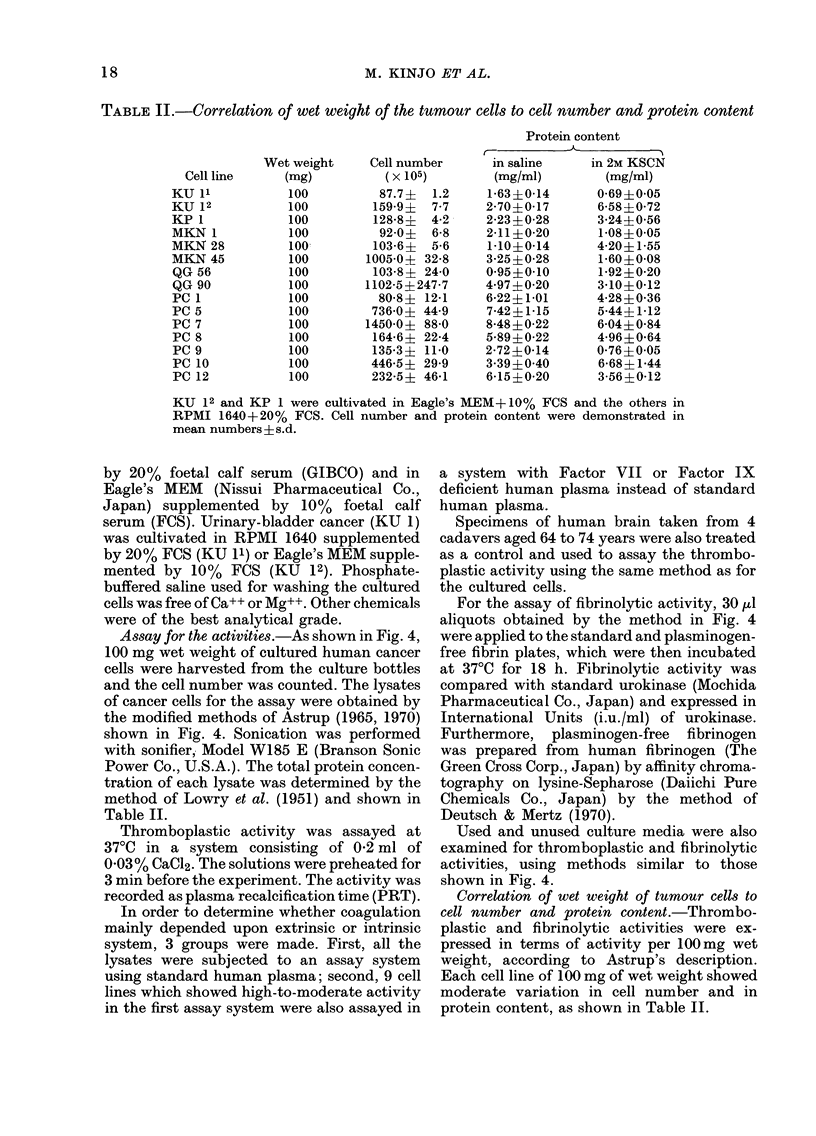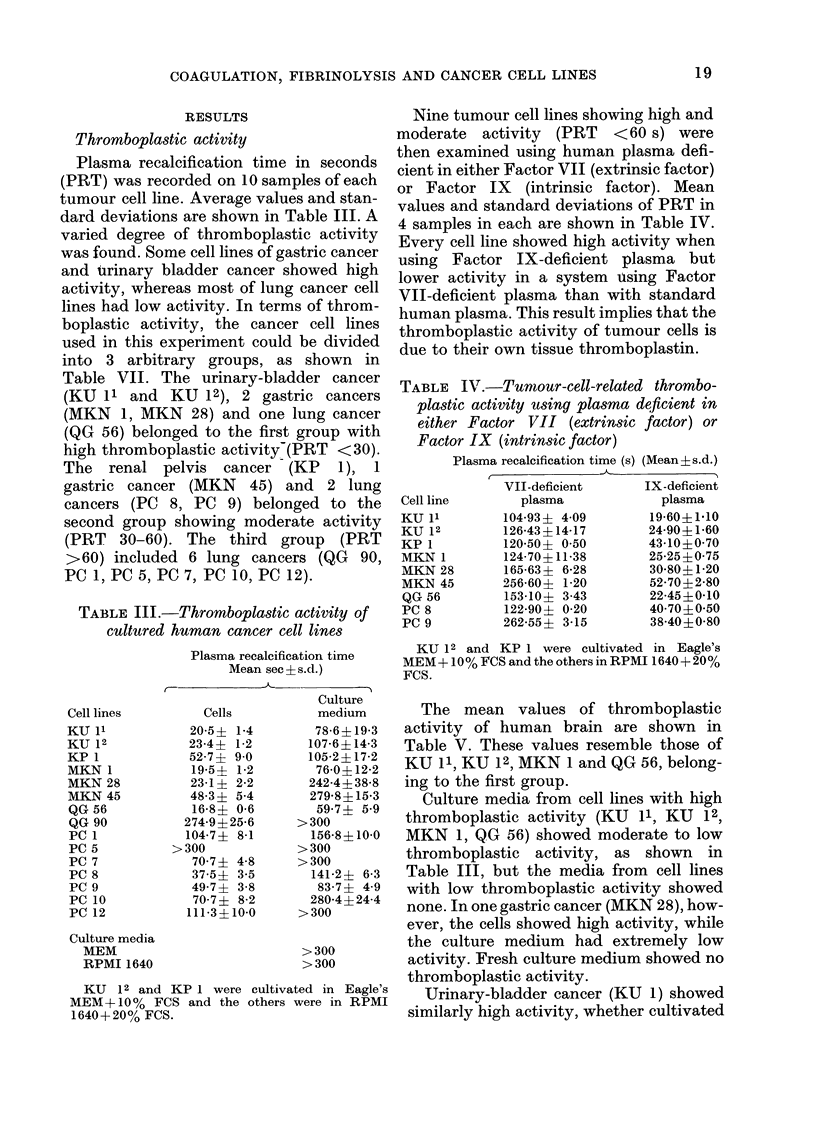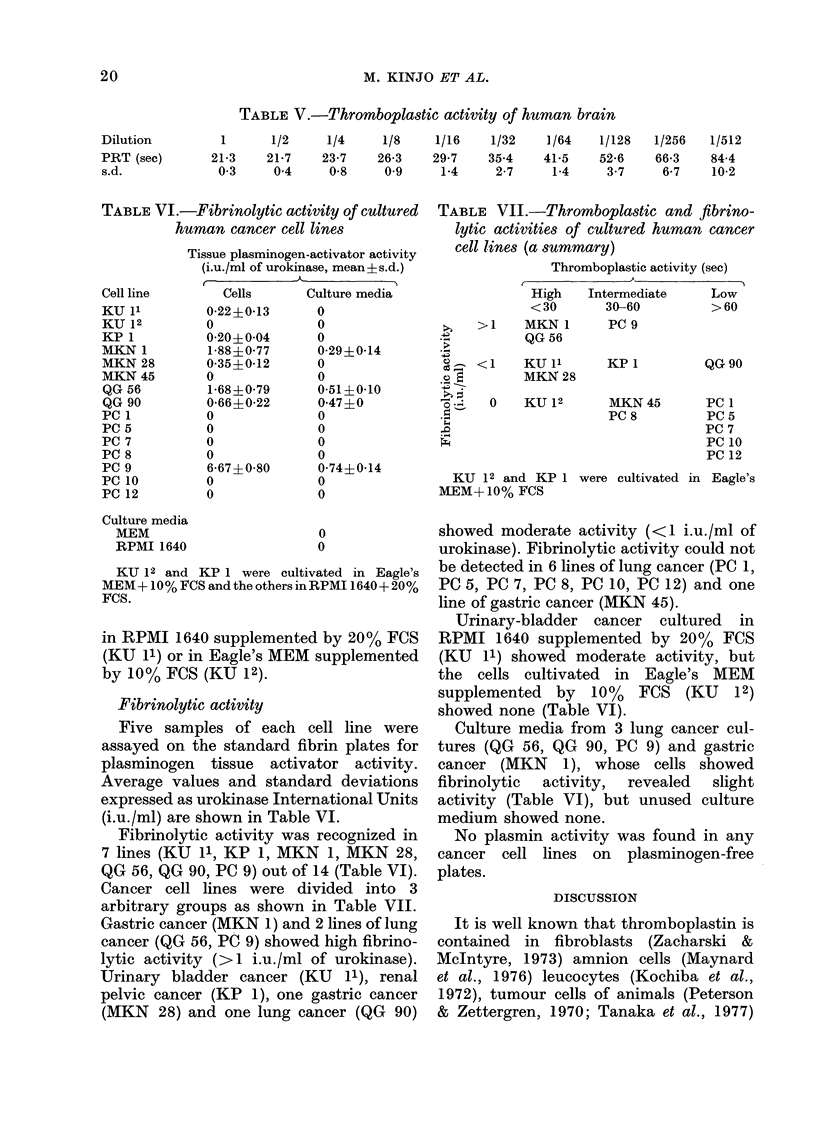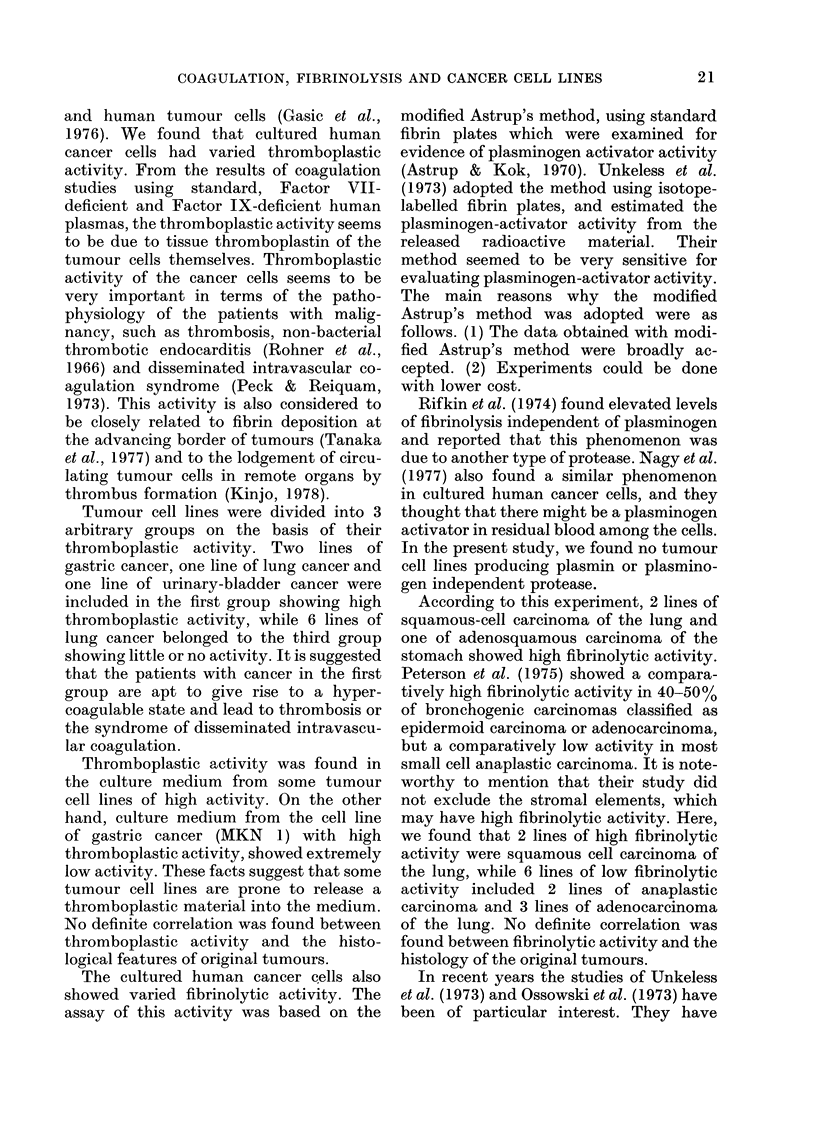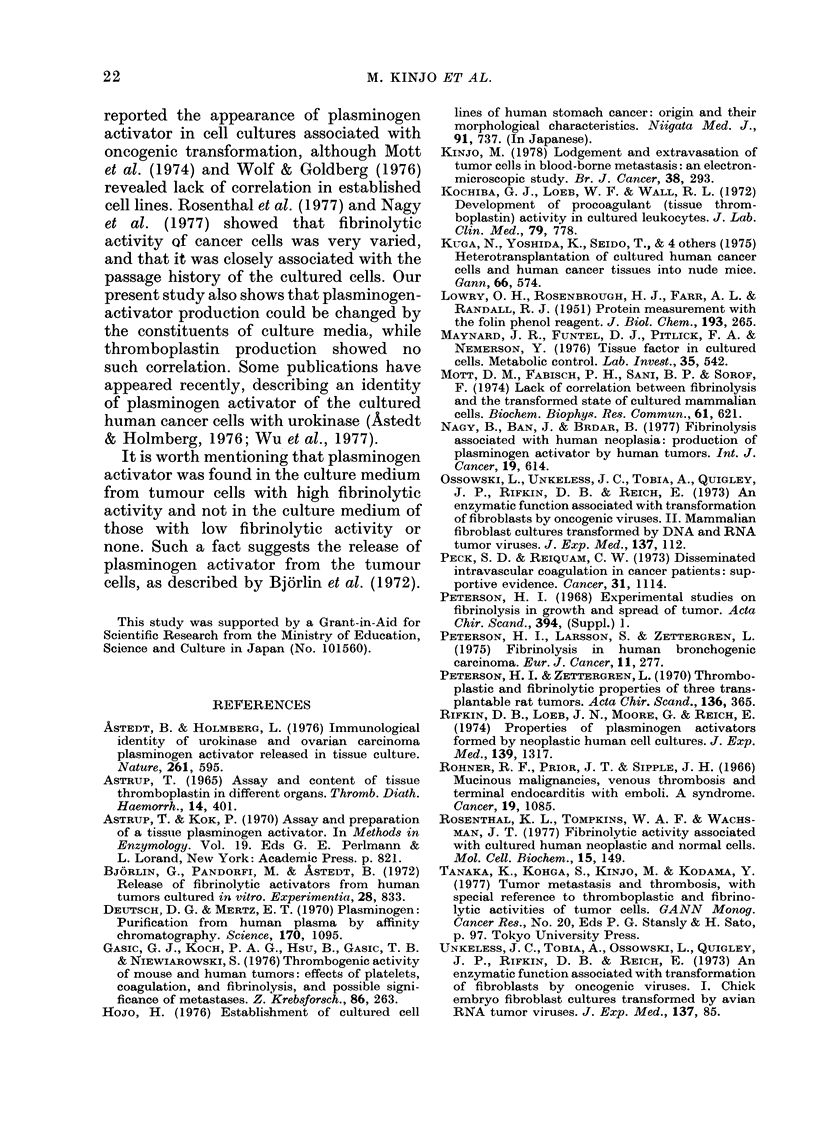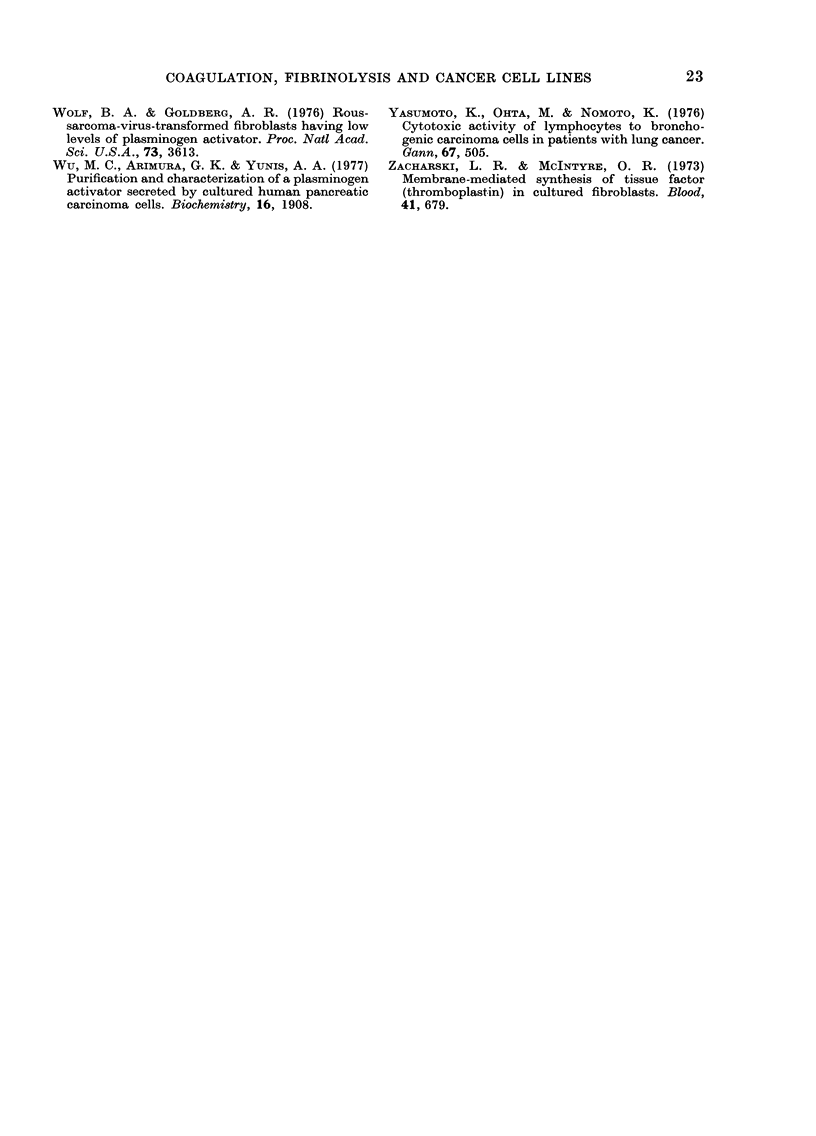# Thromboplastic and fibrinolytic activities of cultured human cancer cell lines.

**DOI:** 10.1038/bjc.1979.3

**Published:** 1979-01

**Authors:** M. Kinjo, K. Oka, S. Naito, S. Kohga, K. Tanaka, S. Oboshi, Y. Hayata, K. Yasumoto

## Abstract

**Images:**


*Br. J. Cancer* (1979) **39**, 15

### THROMBOPLASTIC AND FIBRINOLYTIC ACTIVITIES OF CULTURED HUMAN CANCER CELL LINES

M. KINJO*, K. OKA*, S. NAITO*, S. KOHGA*, K. TANAKA*, S. OBOSHI†, Y. HAYATA‡ AND K. YASUMOTO§

*From the *Department of Pathology, Faculty of Medicine, Kyushu University, Fukuoka, the †Department of Pathology, Faculty of Medicine, Niigata University, Niigata, the ‡Department of Surgery, Tokyo Medical College, Tokyo and the §Division of Thoracic Surgery, Kyushu Cancer Center Hospital, Fukuoka, Japan*

Received 31 March 1978 Accepted 27 September 1978

**Summary.—Thromboplastic and fibrinolytic activities of 14 lines of cultured human cancer cells were estimated by modified Astrup's methods. High tissue thromboplastic activity was found in one line of urinary-bladder cancer, 2 lines of gastric cancer and one line of lung cancer, but no activity was found in 6 lines of lung cancer. High fibrinolytic activity was noted in one line of gastric cancer and 2 lines of lung cancer, but no activity was seen in 6 lines of lung cancer and one line of gastric cancer. No plasmin activity was found. The tumour cell lines could be classified into 3 groups on the basis of the 2 activities. Cancer cell lines could also be classified into 2 groups: with high or low release of thromboplastin into culture media. Fibrinolytic activity was found in the culture media of all cell lines with high fibrinolytic activity. Fibrinolytic activity, but not thromboplastic activity, seemed to be influenced by the constituents of culture media. No definite correlation was found between the 2 activities and the histological types of the parent tumours of the cultured cells.**

THROMBOPLASTIC and fibrinolytic activities of tumour cells themselves are considered to be important factors in the following events: first, thrombosis (Rohner *et al.*, 1966) and disseminated intravascular coagulation syndrome (Peck & Reiquam, 1973) in cancer patients; second, local growth of the tumour at the primary site (Peterson, 1968); third, haematogenous metastases in the form of lodgement and extravasation of arrested tumour cells in remote organs (Tanaka *et al.*, 1977).

Since the reports by Unkeless *et al.* (1973) and Ossowski *et al.* (1973), the correlation between oncogenic transformation of cells and plasminogen activator production in established cell lines has been watched with keen interest. Such a plasminogen activator secreted by cultured human cancer cell lines has also been investigated in terms of purification and characterization of its true nature (Wu *et al.*, 1977).

Thus, much information has now been accumulated on the role of coagulation and fibrinolysis systems of cancer cells in the field of oncology. But the systematic evaluation of thromboplastic and fibrinolytic activities of human cancer cells themselves has not been made.

In the present study, we estimated these activities of 14 lines of human cancer cells *in vitro*, from which thromboplastic material and plasminogen activator originating from the stromal elements of the tumour and/or from the blood are excluded.

### MATERIALS AND METHODS

*Cancer cells.*—The cancer cell lines used in this experiment were composed of one line of urinary bladder cancer (KU 1), one line of renal pelvic cancer (KP 1), 3 lines of gastric cancer (MKN 1, MKN 28, MKN 45) and 9 lines of lung cancer (QG 56, QG 90, PC 1, PC 5, PC 7, PC 8, PC 9, PC 10, PC 12). KU 1 was estab-

FIG. 1.—(a) Histology of parental tumour of QG 90, diagnosed as small-cell anaplastic carcinoma. ×170. (b) Histology of QG 90 transplanted s.c. into nude mouse. Small hyperchromatic tumour cells similar to those in (a) proliferate diffusely in the dermis. ×170.

FIG. 2.—Histology of PC 10 transplanted s.c. into nude mouse. Large tumour cells of squamous-cell carcinoma proliferate in the dermis showing whorl pattern in part. ×170.

FIG. 3.—Histology of PC 12 transplanted s.c. into nude mouse. Squamous-cell carcinoma proliferates in the dermis. Pseudohorn cysts are seen in the upper left corner. ×170.

TABLE I.—*Histological type of original cancers of cultured cell lines*

| | | |
|---|---|---|
| KU 1 | Urinary bladder | Transitional carcinoma |
| KP 1 | Renal pelvis | Anaplastic carcinoma |
| MKN 1 | Stomach | Adenocarcinoma |
| MKN 28 | Stomach | Well differentiated adenocarcinoma |
| MKN 45 | Stomach | Medullary carcinoma |
| QG 56 | Lung | Squamous-cell carcinoma |
| QG 90 | Lung | Small-cell anaplastic carcinoma |
| PC 1 | Lung | Squamous-cell carcinoma |
| PC 5 | Lung | Small-cell anaplastic carcinoma |
| PC 7 | Lung | Poorly differentiated adenocarcinoma |
| PC 8 | Lung | Poorly differentiated adenocarcinoma |
| PC 9 | Lung | Squamous-cell carcinoma |
| PC 10 | Lung | Squamous-cell carcinoma |
| PC 12 | Lung | Papillary adenocarcinoma |

lished at Keio University; MKN 1, MKN 28 and MKN 45 were at Niigata University; PC 1, PC 5, PC 7, PC 8, PC 9, PC 10, PC 12 were at Tokyo Medical College; QG 56, QG 90 were at Kyushu Cancer Centre Hospital. KP 1 was established at Kyushu University. Nine (KU 1, MKN 1, MKN 28, MKN 45, QG 56, PC 1, PC 5, PC 7, PC 8) of the 14 cell lines were described as established cell lines in the literature (Kuga *et al.*, 1975; Yasumoto *et al.*, 1976; Hojo, 1976). The others were proved for their tumorigenicity in nude mice, histological identity between the parent tumours and the transplanted tumours in nude mice (Figs 1, 2 and 3) and progressive growth in *in vitro* culture systems, although they have not been published yet. All the cell lines were cultivated more than 10 passages before the experiment at the Department of Pathology, Faculty of Medicine, Kyushu University. Histological cell type of the original tumours at operation was shown in Table I. The cancer cells were cultivated in RPMI 1640 medium (GIBCO) supplemented

Culture of human cancer cells
↓
Washed twice with PBS (—)*
↓
Suspended in PBS (—) and centrifuged, 1000 rev/min 10 min
↓
Pellet of cancer cells (100 mg wet weight)

THROMBOPLASTIC ACTIVITY ↙

Suspended in 0·9 ml physiological saline
↓
Freeze and thawing
↓
Sonication 30W, 10 sec
↓
Centrifuged, 2000 rev/min 10 min
↓
Supernate
↓
Plasma recalcification time

↘ FIBRINOLYTIC ACTIVITY

Suspended in 3 ml of 2M KSCN
↓
Freeze and thawing
↓
Sonication 30 W, 10 sec and vibration 60 min
↓
Centrifuged, 2000 rev/min 10 min
↓
Adjusted to pH 1 with N HCl
↓
Centrifuged, 2000 rev/min 10 min
↓
Dissolve the precipitate in 2M KSCN
↓
Adjusted to pH 7 with solid $NaHCO_3$
↓
Fibrin plate

FIG. 4.—Assay methods for thromboplastic and fibrinolytic activities of cultured human cancer cells. *PBS without $Ca^{++}$ or $Mg^{++}$.

TABLE II.—*Correlation of wet weight of the tumour cells to cell number and protein content*

| Cell line | Wet weight (mg) | Cell number ($\times 10^5$) | Protein content in saline (mg/ml) | Protein content in 2M KSCN (mg/ml) |
|---|---|---|---|---|
| KU $1^1$ | 100 | 87.7± 1.2 | 1·63±0·14 | 0·69±0·05 |
| KU $1^2$ | 100 | 159·9± 7·7 | 2·70±0·17 | 6·58±0·72 |
| KP 1 | 100 | 128·8± 4·2 | 2·23±0·28 | 3·24±0·56 |
| MKN 1 | 100 | 92·0± 6·8 | 2·11±0·20 | 1·08±0·05 |
| MKN 28 | 100 | 103·6± 5·6 | 1·10±0·14 | 4·20±1·55 |
| MKN 45 | 100 | 1005·0± 32·8 | 3·25±0·28 | 1·60±0·08 |
| QG 56 | 100 | 103·8± 24·0 | 0·95±0·10 | 1·92±0·20 |
| QG 90 | 100 | 1102·5±247·7 | 4·97±0·20 | 3·10±0·12 |
| PC 1 | 100 | 80·8± 12·1 | 6·22±1·01 | 4·28±0·36 |
| PC 5 | 100 | 736·0± 44·9 | 7·42±1·15 | 5·44±1·12 |
| PC 7 | 100 | 1450·0± 88·0 | 8·48±0·22 | 6·04±0·84 |
| PC 8 | 100 | 164·6± 22·4 | 5·89±0·22 | 4·96±0·64 |
| PC 9 | 100 | 135·3± 11·0 | 2·72±0·14 | 0·76±0·05 |
| PC 10 | 100 | 446·5± 29·9 | 3·39±0·40 | 6·68±1·44 |
| PC 12 | 100 | 232·5± 46·1 | 6·15±0·20 | 3·56±0·12 |

KU $1^2$ and KP 1 were cultivated in Eagle's MEM+10% FCS and the others in RPMI 1640+20% FCS. Cell number and protein content were demonstrated in mean numbers±s.d.

by 20% foetal calf serum (GIBCO) and in Eagle's MEM (Nissui Pharmaceutical Co., Japan) supplemented by 10% foetal calf serum (FCS). Urinary-bladder cancer (KU 1) was cultivated in RPMI 1640 supplemented by 20% FCS (KU $1^1$) or Eagle's MEM supplemented by 10% FCS (KU $1^2$). Phosphate-buffered saline used for washing the cultured cells was free of $Ca^{++}$ or $Mg^{++}$. Other chemicals were of the best analytical grade.

*Assay for the activities.*—As shown in Fig. 4, 100 mg wet weight of cultured human cancer cells were harvested from the culture bottles and the cell number was counted. The lysates of cancer cells for the assay were obtained by the modified methods of Astrup (1965, 1970) shown in Fig. 4. Sonication was performed with sonifier, Model W185 E (Branson Sonic Power Co., U.S.A.). The total protein concentration of each lysate was determined by the method of Lowry *et al.* (1951) and shown in Table II.

Thromboplastic activity was assayed at 37°C in a system consisting of 0·2 ml of 0·03% $CaCl_2$. The solutions were preheated for 3 min before the experiment. The activity was recorded as plasma recalcification time (PRT).

In order to determine whether coagulation mainly depended upon extrinsic or intrinsic system, 3 groups were made. First, all the lysates were subjected to an assay system using standard human plasma; second, 9 cell lines which showed high-to-moderate activity in the first assay system were also assayed in a system with Factor VII or Factor IX deficient human plasma instead of standard human plasma.

Specimens of human brain taken from 4 cadavers aged 64 to 74 years were also treated as a control and used to assay the thromboplastic activity using the same method as for the cultured cells.

For the assay of fibrinolytic activity, 30 $\mu$l aliquots obtained by the method in Fig. 4 were applied to the standard and plasminogen-free fibrin plates, which were then incubated at 37°C for 18 h. Fibrinolytic activity was compared with standard urokinase (Mochida Pharmaceutical Co., Japan) and expressed in International Units (i.u./ml) of urokinase. Furthermore, plasminogen-free fibrinogen was prepared from human fibrinogen (The Green Cross Corp., Japan) by affinity chromatography on lysine-Sepharose (Daiichi Pure Chemicals Co., Japan) by the method of Deutsch & Mertz (1970).

Used and unused culture media were also examined for thromboplastic and fibrinolytic activities, using methods similar to those shown in Fig. 4.

*Correlation of wet weight of tumour cells to cell number and protein content.*—Thromboplastic and fibrinolytic activities were expressed in terms of activity per 100 mg wet weight, according to Astrup's description. Each cell line of 100 mg of wet weight showed moderate variation in cell number and in protein content, as shown in Table II.

### RESULTS

### *Thromboplastic activity*

Plasma recalcification time in seconds (PRT) was recorded on 10 samples of each tumour cell line. Average values and standard deviations are shown in Table III. A varied degree of thromboplastic activity was found. Some cell lines of gastric cancer and urinary bladder cancer showed high activity, whereas most of lung cancer cell lines had low activity. In terms of thromboplastic activity, the cancer cell lines used in this experiment could be divided into 3 arbitrary groups, as shown in Table VII. The urinary-bladder cancer (KU $1^1$ and KU $1^2$), 2 gastric cancers (MKN 1, MKN 28) and one lung cancer (QG 56) belonged to the first group with high thromboplastic activity (PRT <30). The renal pelvis cancer (KP 1), 1 gastric cancer (MKN 45) and 2 lung cancers (PC 8, PC 9) belonged to the second group showing moderate activity (PRT 30–60). The third group (PRT >60) included 6 lung cancers (QG 90, PC 1, PC 5, PC 7, PC 10, PC 12).

TABLE III.—*Thromboplastic activity of cultured human cancer cell lines*

| Cell lines | Plasma recalcification time Mean sec ± s.d.) Cells | Culture medium |
|---|---|---|
| KU $1^1$ | 20·5 ± 1·4 | 78·6 ± 19·3 |
| KU $1^2$ | 23·4 ± 1·2 | 107·6 ± 14·3 |
| KP 1 | 52·7 ± 9·0 | 105·2 ± 17·2 |
| MKN 1 | 19·5 ± 1·2 | 76·0 ± 12·2 |
| MKN 28 | 23·1 ± 2·2 | 242·4 ± 38·8 |
| MKN 45 | 48·3 ± 5·4 | 279·8 ± 15·3 |
| QG 56 | 16·8 ± 0·6 | 59·7 ± 5·9 |
| QG 90 | 274·9 ± 25·6 | >300 |
| PC 1 | 104·7 ± 8·1 | 156·8 ± 10·0 |
| PC 5 | >300 | >300 |
| PC 7 | 70·7 ± 4·8 | >300 |
| PC 8 | 37·5 ± 3·5 | 141·2 ± 6·3 |
| PC 9 | 49·7 ± 3·8 | 83·7 ± 4·9 |
| PC 10 | 70·7 ± 8·2 | 280·4 ± 24·4 |
| PC 12 | 111·3 ± 10·0 | >300 |
| Culture media | | |
| MEM | | >300 |
| RPMI 1640 | | >300 |

KU $1^2$ and KP 1 were cultivated in Eagle's MEM + 10% FCS and the others were in RPMI 1640 + 20% FCS.

Nine tumour cell lines showing high and moderate activity (PRT <60 s) were then examined using human plasma deficient in either Factor VII (extrinsic factor) or Factor IX (intrinsic factor). Mean values and standard deviations of PRT in 4 samples in each are shown in Table IV. Every cell line showed high activity when using Factor IX-deficient plasma but lower activity in a system using Factor VII-deficient plasma than with standard human plasma. This result implies that the thromboplastic activity of tumour cells is due to their own tissue thromboplastin.

TABLE IV.—*Tumour-cell-related thromboplastic activity using plasma deficient in either Factor VII (extrinsic factor) or Factor IX (intrinsic factor)*

| Cell line | Plasma recalcification time (s) (Mean ± s.d.) VII-deficient plasma | IX-deficient plasma |
|---|---|---|
| KU $1^1$ | 104·93 ± 4·09 | 19·60 ± 1·10 |
| KU $1^2$ | 126·43 ± 14·17 | 24·90 ± 1·60 |
| KP 1 | 120·50 ± 0·50 | 43·10 ± 0·70 |
| MKN 1 | 124·70 ± 11·38 | 25·25 ± 0·75 |
| MKN 28 | 165·63 ± 6·28 | 30·80 ± 1·20 |
| MKN 45 | 256·60 ± 1·20 | 52·70 ± 2·80 |
| QG 56 | 153·10 ± 3·43 | 22·45 ± 0·10 |
| PC 8 | 122·90 ± 0·20 | 40·70 ± 0·50 |
| PC 9 | 262·55 ± 3·15 | 38·40 ± 0·80 |

KU $1^2$ and KP 1 were cultivated in Eagle's MEM + 10% FCS and the others in RPMI 1640 + 20% FCS.

The mean values of thromboplastic activity of human brain are shown in Table V. These values resemble those of KU $1^1$, KU $1^2$, MKN 1 and QG 56, belonging to the first group.

Culture media from cell lines with high thromboplastic activity (KU $1^1$, KU $1^2$, MKN 1, QG 56) showed moderate to low thromboplastic activity, as shown in Table III, but the media from cell lines with low thromboplastic activity showed none. In one gastric cancer (MKN 28), however, the cells showed high activity, while the culture medium had extremely low activity. Fresh culture medium showed no thromboplastic activity.

Urinary-bladder cancer (KU 1) showed similarly high activity, whether cultivated

TABLE V.—*Thromboplastic activity of human brain*

| Dilution | 1 | 1/2 | 1/4 | 1/8 | 1/16 | 1/32 | 1/64 | 1/128 | 1/256 | 1/512 |
|---|---|---|---|---|---|---|---|---|---|---|
| PRT (sec) | 21·3 | 21·7 | 23·7 | 26·3 | 29·7 | 35·4 | 41·5 | 52·6 | 66·3 | 84·4 |
| s.d. | 0·3 | 0·4 | 0·8 | 0·9 | 1·4 | 2·7 | 1·4 | 3·7 | 6·7 | 10·2 |

TABLE VI.—*Fibrinolytic activity of cultured human cancer cell lines*

| | Tissue plasminogen-activator activity (i.u./ml of urokinase, mean ± s.d.) | |
|---|---|---|
| Cell line | Cells | Culture media |
| KU 1[1] | 0·22±0·13 | 0 |
| KU 1[2] | 0 | 0 |
| KP 1 | 0·20±0·04 | 0 |
| MKN 1 | 1·88±0·77 | 0·29±0·14 |
| MKN 28 | 0·35±0·12 | 0 |
| MKN 45 | 0 | 0 |
| QG 56 | 1·68±0·79 | 0·51±0·10 |
| QG 90 | 0·66±0·22 | 0·47±0 |
| PC 1 | 0 | 0 |
| PC 5 | 0 | 0 |
| PC 7 | 0 | 0 |
| PC 8 | 0 | 0 |
| PC 9 | 6·67±0·80 | 0·74±0·14 |
| PC 10 | 0 | 0 |
| PC 12 | 0 | 0 |
| Culture media | | |
| MEM | | 0 |
| RPMI 1640 | | 0 |

KU 1[2] and KP 1 were cultivated in Eagle's MEM+10% FCS and the others in RPMI 1640+20% FCS.

TABLE VII.—*Thromboplastic and fibrinolytic activities of cultured human cancer cell lines* (*a summary*)

| Fibrinolytic activity (i.u./ml) | Thromboplastic activity (sec): High <30 | Intermediate 30–60 | Low >60 |
|---|---|---|---|
| >1 | MKN 1<br>QG 56 | PC 9 | |
| <1 | KU 1[1]<br>MKN 28 | KP 1 | QG 90 |
| 0 | KU 1[2] | MKN 45<br>PC 8 | PC 1<br>PC 5<br>PC 7<br>PC 10<br>PC 12 |

KU 1[2] and KP 1 were cultivated in Eagle's MEM+10% FCS

in RPMI 1640 supplemented by 20% FCS (KU 1[1]) or in Eagle's MEM supplemented by 10% FCS (KU 1[2]).

*Fibrinolytic activity*

Five samples of each cell line were assayed on the standard fibrin plates for plasminogen tissue activator activity. Average values and standard deviations expressed as urokinase International Units (i.u./ml) are shown in Table VI.

Fibrinolytic activity was recognized in 7 lines (KU 1[1], KP 1, MKN 1, MKN 28, QG 56, QG 90, PC 9) out of 14 (Table VI). Cancer cell lines were divided into 3 arbitrary groups as shown in Table VII. Gastric cancer (MKN 1) and 2 lines of lung cancer (QG 56, PC 9) showed high fibrinolytic activity (>1 i.u./ml of urokinase). Urinary bladder cancer (KU 1[1]), renal pelvic cancer (KP 1), one gastric cancer (MKN 28) and one lung cancer (QG 90) showed moderate activity (<1 i.u./ml of urokinase). Fibrinolytic activity could not be detected in 6 lines of lung cancer (PC 1, PC 5, PC 7, PC 8, PC 10, PC 12) and one line of gastric cancer (MKN 45).

Urinary-bladder cancer cultured in RPMI 1640 supplemented by 20% FCS (KU 1[1]) showed moderate activity, but the cells cultivated in Eagle's MEM supplemented by 10% FCS (KU 1[2]) showed none (Table VI).

Culture media from 3 lung cancer cultures (QG 56, QG 90, PC 9) and gastric cancer (MKN 1), whose cells showed fibrinolytic activity, revealed slight activity (Table VI), but unused culture medium showed none.

No plasmin activity was found in any cancer cell lines on plasminogen-free plates.

### DISCUSSION

It is well known that thromboplastin is contained in fibroblasts (Zacharski & McIntyre, 1973) amnion cells (Maynard *et al.*, 1976) leucocytes (Kochiba *et al.*, 1972), tumour cells of animals (Peterson & Zettergren, 1970; Tanaka *et al.*, 1977)

and human tumour cells (Gasic *et al.*, 1976). We found that cultured human cancer cells had varied thromboplastic activity. From the results of coagulation studies using standard, Factor VII-deficient and Factor IX-deficient human plasmas, the thromboplastic activity seems to be due to tissue thromboplastin of the tumour cells themselves. Thromboplastic activity of the cancer cells seems to be very important in terms of the pathophysiology of the patients with malignancy, such as thrombosis, non-bacterial thrombotic endocarditis (Rohner *et al.*, 1966) and disseminated intravascular coagulation syndrome (Peck & Reiquam, 1973). This activity is also considered to be closely related to fibrin deposition at the advancing border of tumours (Tanaka *et al.*, 1977) and to the lodgement of circulating tumour cells in remote organs by thrombus formation (Kinjo, 1978).

Tumour cell lines were divided into 3 arbitrary groups on the basis of their thromboplastic activity. Two lines of gastric cancer, one line of lung cancer and one line of urinary-bladder cancer were included in the first group showing high thromboplastic activity, while 6 lines of lung cancer belonged to the third group showing little or no activity. It is suggested that the patients with cancer in the first group are apt to give rise to a hypercoagulable state and lead to thrombosis or the syndrome of disseminated intravascular coagulation.

Thromboplastic activity was found in the culture medium from some tumour cell lines of high activity. On the other hand, culture medium from the cell line of gastric cancer (MKN 1) with high thromboplastic activity, showed extremely low activity. These facts suggest that some tumour cell lines are prone to release a thromboplastic material into the medium. No definite correlation was found between thromboplastic activity and the histological features of original tumours.

The cultured human cancer cells also showed varied fibrinolytic activity. The assay of this activity was based on the modified Astrup's method, using standard fibrin plates which were examined for evidence of plasminogen activator activity (Astrup & Kok, 1970). Unkeless *et al.* (1973) adopted the method using isotope-labelled fibrin plates, and estimated the plasminogen-activator activity from the released radioactive material. Their method seemed to be very sensitive for evaluating plasminogen-activator activity. The main reasons why the modified Astrup's method was adopted were as follows. (1) The data obtained with modified Astrup's method were broadly accepted. (2) Experiments could be done with lower cost.

Rifkin *et al.* (1974) found elevated levels of fibrinolysis independent of plasminogen and reported that this phenomenon was due to another type of protease. Nagy *et al.* (1977) also found a similar phenomenon in cultured human cancer cells, and they thought that there might be a plasminogen activator in residual blood among the cells. In the present study, we found no tumour cell lines producing plasmin or plasminogen independent protease.

According to this experiment, 2 lines of squamous-cell carcinoma of the lung and one of adenosquamous carcinoma of the stomach showed high fibrinolytic activity. Peterson *et al.* (1975) showed a comparatively high fibrinolytic activity in 40–50% of bronchogenic carcinomas classified as epidermoid carcinoma or adenocarcinoma, but a comparatively low activity in most small cell anaplastic carcinoma. It is noteworthy to mention that their study did not exclude the stromal elements, which may have high fibrinolytic activity. Here, we found that 2 lines of high fibrinolytic activity were squamous cell carcinoma of the lung, while 6 lines of low fibrinolytic activity included 2 lines of anaplastic carcinoma and 3 lines of adenocarcinoma of the lung. No definite correlation was found between fibrinolytic activity and the histology of the original tumours.

In recent years the studies of Unkeless *et al.* (1973) and Ossowski *et al.* (1973) have been of particular interest. They have reported the appearance of plasminogen activator in cell cultures associated with oncogenic transformation, although Mott *et al.* (1974) and Wolf & Goldberg (1976) revealed lack of correlation in established cell lines. Rosenthal *et al.* (1977) and Nagy *et al.* (1977) showed that fibrinolytic activity of cancer cells was very varied, and that it was closely associated with the passage history of the cultured cells. Our present study also shows that plasminogen-activator production could be changed by the constituents of culture media, while thromboplastin production showed no such correlation. Some publications have appeared recently, describing an identity of plasminogen activator of the cultured human cancer cells with urokinase (Åstedt & Holmberg, 1976; Wu *et al.*, 1977).

It is worth mentioning that plasminogen activator was found in the culture medium from tumour cells with high fibrinolytic activity and not in the culture medium of those with low fibrinolytic activity or none. Such a fact suggests the release of plasminogen activator from the tumour cells, as described by Björlin *et al.* (1972).

This study was supported by a Grant-in-Aid for Scientific Research from the Ministry of Education, Science and Culture in Japan (No. 101560).

REFERENCES

ÅSTEDT, B. & HOLMBERG, L. (1976) Immunological identity of urokinase and ovarian carcinoma plasminogen activator released in tissue culture. *Nature*, **261**, 595.

ASTRUP, T. (1965) Assay and content of tissue thromboplastin in different organs. *Thromb. Diath. Haemorrh.*, **14**, 401.

ASTRUP, T. & KOK, P. (1970) Assay and preparation of a tissue plasminogen activator. In *Methods in Enzymology*. Vol. 19. Eds G. E. Perlmann & L. Lorand, New York: Academic Press. p. 821.

BJÖRLIN, G., PANDORFI, M. & ÅSTEDT, B. (1972) Release of fibrinolytic activators from human tumors cultured *in vitro*. *Experimentia*, **28**, 833.

DEUTSCH, D. G. & MERTZ, E. T. (1970) Plasminogen: Purification from human plasma by affinity chromatography. *Science*, **170**, 1095.

GASIC, G. J., KOCH, P. A. G., HSU, B., GASIC, T. B. & NIEWIAROWSKI, S. (1976) Thrombogenic activity of mouse and human tumors: effects of platelets, coagulation, and fibrinolysis, and possible significance of metastases. *Z. Krebsforsch.*, **86**, 263.

HOJO, H. (1976) Establishment of cultured cell lines of human stomach cancer: origin and their morphological characteristics. *Niigata Med. J.*, **91**, 737. (In Japanese).

KINJO, M. (1978) Lodgement and extravasation of tumor cells in blood-borne metastasis: an electron-microscopic study. *Br. J. Cancer*, **38**, 293.

KOCHIBA, G. J., LOEB, W. F. & WALL, R. L. (1972) Development of procoagulant (tissue thromboplastin) activity in cultured leukocytes. *J. Lab. Clin. Med.*, **79**, 778.

KUGA, N., YOSHIDA, K., SEIDO, T., & 4 others (1975) Heterotransplantation of cultured human cancer cells and human cancer tissues into nude mice. *Gann*, **66**, 574.

LOWRY, O. H., ROSENBROUGH, H. J., FARR, A. L. & RANDALL, R. J. (1951) Protein measurement with the folin phenol reagent. *J. Biol. Chem.*, **193**, 265.

MAYNARD, J. R., FUNTEL, D. J., PITLICK, F. A. & NEMERSON, Y. (1976) Tissue factor in cultured cells. Metabolic control. *Lab. Invest.*, **35**, 542.

MOTT, D. M., FABISCH, P. H., SANI, B. P. & SOROF, F. (1974) Lack of correlation between fibrinolysis and the transformed state of cultured mammalian cells. *Biochem. Biophys. Res. Commun.*, **61**, 621.

NAGY, B., BAN, J. & BRDAR, B. (1977) Fibrinolysis associated with human neoplasia: production of plasminogen activator by human tumors. *Int. J. Cancer*, **19**, 614.

OSSOWSKI, L., UNKELESS, J. C., TOBIA, A., QUIGLEY, J. P., RIFKIN, D. B. & REICH, E. (1973) An enzymatic function associated with transformation of fibroblasts by oncogenic viruses. II. Mammalian fibroblast cultures transformed by DNA and RNA tumor viruses. *J. Exp. Med.*, **137**, 112.

PECK, S. D. & REIQUAM, C. W. (1973) Disseminated intravascular coagulation in cancer patients: supportive evidence. *Cancer*, **31**, 1114.

PETERSON, H. I. (1968) Experimental studies on fibrinolysis in growth and spread of tumor. *Acta Chir. Scand.*, **394**, (Suppl.) 1.

PETERSON, H. I., LARSSON, S. & ZETTERGREN, L. (1975) Fibrinolysis in human bronchogenic carcinoma. *Eur. J. Cancer*, **11**, 277.

PETERSON, H. I. & ZETTERGREN, L. (1970) Thromboplastic and fibrinolytic properties of three transplantable rat tumors. *Acta Chir. Scand.*, **136**, 365.

RIFKIN, D. B., LOEB, J. N., MOORE, G. & REICH, E. (1974) Properties of plasminogen activators formed by neoplastic human cell cultures. *J. Exp. Med.*, **139**, 1317.

ROHNER, R. F., PRIOR, J. T. & SIPPLE, J. H. (1966) Mucinous malignancies, venous thrombosis and terminal endocarditis with emboli. A syndrome. *Cancer*, **19**, 1085.

ROSENTHAL, K. L., TOMPKINS, W. A. F. & WACHSMAN, J. T. (1977) Fibrinolytic activity associated with cultured human neoplastic and normal cells. *Mol. Cell. Biochem.*, **15**, 149.

TANAKA, K., KOHGA, S., KINJO, M. & KODAMA, Y. (1977) Tumor metastasis and thrombosis, with special reference to thromboplastic and fibrinolytic activities of tumor cells. *GANN Monog. Cancer Res.*, No. 20, Eds P. G. Stansly & H. Sato, p. 97. Tokyo University Press.

UNKELESS, J. C., TOBIA, A., OSSOWSKI, L., QUIGLEY, J. P., RIFKIN, D. B. & REICH, E. (1973) An enzymatic function associated with transformation of fibroblasts by oncogenic viruses. I. Chick embryo fibroblast cultures transformed by avian RNA tumor viruses. *J. Exp. Med.*, **137**, 85.

WOLF, B. A. & GOLDBERG, A. R. (1976) Rous-sarcoma-virus-transformed fibroblasts having low levels of plasminogen activator. *Proc. Natl Acad. Sci. U.S.A.*, **73**, 3613.

WU, M. C., ARIMURA, G. K. & YUNIS, A. A. (1977) Purification and characterization of a plasminogen activator secreted by cultured human pancreatic carcinoma cells. *Biochemistry*, **16**, 1908.

YASUMOTO, K., OHTA, M. & NOMOTO, K. (1976) Cytotoxic activity of lymphocytes to bronchogenic carcinoma cells in patients with lung cancer. *Gann*, **67**, 505.

ZACHARSKI, L. R. & MCINTYRE, O. R. (1973) Membrane-mediated synthesis of tissue factor (thromboplastin) in cultured fibroblasts. *Blood*, **41**, 679.